# A Plea for Conservatism

**Published:** 1891-09

**Authors:** W. C. Barrett

**Affiliations:** Buffalo, N. Y.


AMERICAN JOURNAL

—OF—

DENTAL SCIENCE.

VOL. XXV. BALTIMORE, SEPTEMBER, 1891. NO. 5.

ARTICLE I.

### A PLEA FOR CONSERVATISM.

BY W. C. BARRETT, M. D., D. D. S., BUFFALO, N. Y.

Read before the American Dental Association, Saratoga Springs, N. Y. Reprinted from advance sheets of the *Dental Cosmos.*

There are few men who possess minds so evenly balanced that they are enabled to view a subject from all the different standpoints, and, taking into consideration every factor that enters into the sum of the whole, deduce therefrom a fair and judicial estimate of the relative importance of each. One individual element assumes so close a relation to us that we see nothing else. A single angle presents itself to the vision, and gets so near as completely to shut out all view of other proportions, just as a finger's-end may be so close to the eye as to eclipse the whole world beside. This is especially true in all branches of medicince. There are such an infinite number of elements entering into the problem of pathological disturbances, each of which must in turn be taken up singly and carefully examined, that there is little wonder that every new agent presented should absorb attention for the time being, and monopolize a mind not sufficiently capacious to entertain more than one idea at a time. One single part of a great whole becomes so exaggerated by exclusive consideration that it engrosses the whole field, and everything else is lost to sight. Thus, one afflicted with some obscure, recondite, disease, may apply in succession to all the medical specialists, and in turn be told that the source of his illness is in the lungs, the throat, the stomach, the nerves, the genitalia, the eyes, or the teeth. And the opinion will in each case be an honest one, for constant contemplation of one organ, or set of organs, has so exaggerated its importance that, remotely or immediately, they find it connected with every physical aberration.

When chemistry emerged from the dark ages of catalysis and spontaneous transmutation or change, and when was first observed what should have been known at the outset, that every chemical transition was the direct result of an active cause; when man obtained his initial glimpse into that wonderful microscopic world which so greatly transcends the microscopic in extent and interest; when he began to comprehend the molecular disturbances wrought through the proliferation of the vegetable fungi, and to get a little inkling of what was the true nature of those previously mysterious processes called fermentation and putrefaction, if he were the enthusiast that discoverers usually are, he imagined that the whole arcana of nature were now laid bare. Here was a new world of unknown vastness, and the influence of it upon the organic world of which its denizens were parasites was as yet unlimited by actual discovery, and imagination ran riot in the possibilities. These minute organisms might be the so long hidden cause of the unknown. Here might be found the key that would unlock the mysteries of nature. The cosmic egg was hatched. Microbes managed matter. Bacteria dominated disease, and pathology was but another name for a ferment. Corns and consumption were alike due to some specific micro-organism. The whole duty of man, professionally, was to get at the microbe and to destroy him, and then hermetically to seal up the tissues against further infection. Everything else was lost to sight by many of the so-called progressive physicians, and septicism and antiseptics alone ruled the hour.

In medicine the errors into which an unrestrained enthusiasm led such great men as Koch and Lister, and even Pasteur himself, have brought about something of a reaction, and it has been learned that every other study cannot be abandoned for the bacteriological laboratory, great and important as that department is. In dentistry we are slowly approaching the same point. It was with difficulty that the great and sublime truth of which Miller first gave us a clear conception secured fair consideration. I well remember, and so will some of you, when in 1884, only seven years ago, this Association met in this very place, and when upon this floor I attempted to urge some of his views, I, with my friend Daboll, was fairly hooted down. We were howled at, and the wicked did gnash their teeth at us. Every time I opened my mouth one prominent member stopped it by hoarsely bawling, "I call the gentleman to order." But truth has made its way, and now the trouble is that the pendulum has swung to the opposite extremity, and upon this same floor I feel myself compelled to enter a protest against what has assumed the proportions of a craze. The microbe haunts us, and infinitely small though it be, it is large enough to eclipse every other factor. We are now as obscure in the clouds as we were before in the caves. Climbing out of the cellar of ignorance, we have not stopped at the first floor of reason, but have rushed into the garret of fanaticism and zealotry.

Let me not be misapprehended. I am not underrating the importance of a thorough study of microscopical biology, nor would I for a moment depricate the influence of microbes in oral pathological conditions. But I do deprecate the idea that there is nothing else to be considered, and that antiseptics and germicides are the only remedies which our pharmacopœias should contain. I protest against the assumption that because Miller, as the result of his scientific investigations, proves that there is no special remedy that will insure an entire aseptic condition of the oral cavity, and that tooth-decay cannot be entirely prevented, that therefore all the splendid results of his years of investigation are barren and useless.

In a late journal a dentist insists that nothing has been gained in the last decade, because teeth still need to be filled, and alveolar abscess yet occasionally occurs. Look back, you gentlemen who have kept pace with the onward march of progress,—look back and see what was the condition of dental pathological knowledge in 1881. Have we no reason to feel encouraged? Are we not now to a greater extent the masters of disease? Does not the dentist who carefully and discriminatingly uses the knowledge of to-day, adding to it the nice manipulation upon which the practitioner of ten years ago depended,—does he not succeed the better for it?

The great trouble is that many men cannot imbibe a single draught of knowledge without becoming craniologically intoxicated. A single large idea overstimulates their intelligence, and they lose mental balance. There is something more than mere metrical jingle in the famous stanze from Pope,—

"A little learning is a dangerous thing,
Drink deep, or taste not the Pierian spring;
There shallow draughts intoxicate the brain,
And drinking largely sobers us again."

Intelligent and enthusiastic dentists, who have the ear of their confreres, have discoursed learnedly upon microbes and ferments, upon germicides and detergents, upon coagulants and non-coagulants, while the younger men, who were perhaps taking their first sip of the fascinating draught, or at least with less of experience and knowledge, listened with open ears and wondering eyes. Nothing was said of other conditions than that of septicism, because the speaker took it for granted that all were as well informed as himself, and that they would add the modifying provisions. A single factor seemed to be raised to a dominating position, and remedies new even to the chemist were specifically recommended, without a fair comprehension of their properties. Is it any wonder that in following such teaching mortifying failures resulted?

How long is it since immediate root-filling was a kind of craze, the result of just such enthusiastic but injudicious teaching? A new idea was allowed to run away with judgment. Young men ran off from the meetings, like newly-hatched chicks with half the shell upon their backs, and essayed the new practice until sad experience taught them that there were other elements to be considered, and that the dental millennium had not yet arrived. Some of them have sat in sackcloth and ashes ever since, giving dental societies a wide berth, and anathematizing those whom they believe to be false teachers, blind leaders of the blind, and all in the ditch together.

It seems to me time that we begin to consider the subject of antiseptic dental treatment from a more rational standpoint. Microbes cannot be utterly excluded from the mouth. They will penetrate cavities of decay despite the greatest precautions. Utter asepticism in any portion of the human economy is a dream that has no foundation in fact. At the International Medical Congress in London, in 1881, Sir Joseph Lister, who has given his name to a method of operative procedure, announced that the horrors of surgical interference were abolished, and that by means of the carbolic spray it was possible to operate under entirely aseptic conditions. But Zeigler, and Bantock, and Tait secured even greater success than did Lister, and they spurned his spray, being only sedulously careful as to cleanliness and irrigation. At the Congress held in Berlin last summer, we who had listened to Professor Lister with so much suppressed emotion only nine years before, were astounded to hear him now say, "As regards the spray, I feel ashamed that I should ever have recommended it for the purpose of destroying the microbes of the air."

The practice recommended by some of our most intelligent dentists in the treatment of root-canals, it seems to me, looks toward the entire exclusion of microbes. Everything is managed with this end in view. The utmost stress is laid upon the most minute particulars, while other needed precautions are neglected. In cases of alveolar abscess we hear of nothing save detergents and antiseptics. It seems to be taken for granted that if microbes can be exterminated, nothing else need be done. In the capping of exposed pulps asepticism is made the only condition of success.

I wish again to say that I am not urging indifference to antiseptic treatment. I believe it to be absolutely essential to successful practice. But I do not think that it is the sole factor, and the only thing to be looked after. I do not believe that the entire absence of all organisms can ever be assured. I do not hold it possible to secure complete aseptic conditions by any external means or agents. We cannot control the process of nature; we can only influence them to a certain degree. We cannot, for instance, furnish the proximate principles of the body from without. We can only supply the proper pabulum, and from that allow the nutritive system to select and prepare its own material. The attempt to harden the bones or the teeth by giving phosphate of lime is a hopeless task, because it is not in accordance with physiological law, and it is quite as absurd to undertake the extermination from the tissues and organs of the body of all the micro-organisms, whether of a pathogenic or non-pathogenic nature. The oral cavity is never without microbes of various kinds.

The multiplication of remedies and the duplication and reduplication of methods will not compass the impossible. The more complicated the process employed, the greater the opportunities for error. The absolute sterilization of dental instruments is impracticable. We may approximate it, but it is hopeless to attempt to destroy all spores without ruining the instrument. The germs of different fungi are too numerous and too widely diffused to be entirely eluded. Wherever air enters, there goes the possibility of infection, and air cannot be utterly excluded. Within the tissues, diffused throughout the blood, penetrating everywhere, the spores of the fungi are found, in health as well as in disease. There is no immunity from infection when an organ or a tissue shall have taken on a condition of atony. Man may fly to the uttermost parts of the earth, but there shall he meet with the destruction that wasteth at noonday. The microbe hath all lands for his own, and it is useless to attempt to escape his presence.

Does this absolve us from all attempts at asepticism? By no means. The very fact of the universality of the microbe should stimulate us to greater precautions. That instruments may not be made absolutely sterile, is no excuse for the almost universal and criminal neglect of proper precautions. How many operators keep a sterilizing apparatus and faithfully use it? It is of no material use merely to dip a point into some solution and then call it sterilized. How many have a special apparatus for the purpose? And yet no man should be permitted to practice without something of the kind. While we cannot utterly banish micro-organisms, we have no right to be inoculating with specific pathogenic bacteria. I have seen many cases in which a previously healthy mouth became diseased after the filling of a tooth by some careless or ignorant dentist. I have under treatment now a very bad case of localized stomatitis, the result of the lack of proper and decent care on the part of a dentist. A beautiful and refined young lady was undoubtedly inoculated from unsterilized instruments that had been used in an affected mouth. We never know how susceptible an apparently healthy patient may be to infection of some pathogenic organism. Even animals, not domesticated, may have such a condition of atony as to subject them to the action of microbes from which it is usually supposed that they have immunity. I have the skull of an old male gorilla which had a very bad alveolar abscess, the result of caries of the teeth, and I have another skull of a female gorilla that suffered from undoubted pyorrhœa. I also have a case of exaggerated antral abscess in a gorilla. You know that this animal has never yet been kept in a state of captivity.

In a condition of entire health nature has made provision for the destruction of invading bacteria. Whether the theories of Metchnikoff be true, and the peculiar power to digest and devour them rests in the leucocytes of the blood,or whether microbicidic action may be found in some other function, we cannot now positively say. But we do know that until the tone of the system be in some way depressed, there is immunity from the usual pathogenic organism. That the spores of different ferments vary in virulence is of course a fact, but that does not affect the truth of the principle.

A tooth that has lost its pulp cannot be said to be in a normal condition. Yet in the healthy mouth, if proper precautions have been taken and if the right mechanical measures for its protection shall have been employed, we know that it is secure against the attack of disease under ordinary circumstances. We know that in many instances a pulp has been destroyed in the most slovenly way imaginable, without any regard even for decent cleanliness, a filling has been inserted over the devitalized pulp without even an attempt to remove the contents of the root-canal, and it has remained in an apparent good condition for years. We know that until within a comparatively short time the roots of teeth were filled without antiseptic precautions, because the condition was not then comprehended, and yet they were sometimes well saved. Westcott and Dwinelle, and their compeers, filled nerve canals before microbes and antiseptics were dreamed of, and they did it with success, too. Antiseptic treatment is not then always an essential. But Westcott and Dwinelle were not able to save as large a number of cases, nor were they with the incomplete comprehension of pathology which belonged to the fifth and sixth decades of the century, warranted in attempting the salvation of a class of teeth which, in the light of the further knowledge of this tenth decade, we do not hesitate to fill.

Thirty years ago there were very few who attempted the filling of root canals, for the men who were able to make delicate manipulation take the place of pathological knowledge were limited in number. Now almost every dentist who reads the journals attempts root treatment, depending upon following the methods laid down by those who pose as teachers. And it is just at this point that I desire to enter my protest against the half-teaching of the journals and societies when, affecting to discuss the whole of a given subject, only a single aspect of it is considered, and that is elevated into a matter of supreme, sole importance. I do not believe that it is sufficient that the cavity of decay should be deluged with detergents, saturated with germicides, aud soaked with antiseptics. It is not enough that saliva should be excluded because of danger from infection. There are other reasons for it. The operation must be skillfully performed in yet another sense, and the manipulation must be perfect. The tissues about the tooth must not be injured by mangling blunders, any more than that they should be removed as far as possible from danger of septic infection. The filling should be judiciously chosen and properly inserted. The appropriate remedies to insure the restoration of the tissues should be employed. No use of germicides can atone for the lack of these and other manipulative measures. While septic conditions should be carefully looked after, even greater stress should be placed on hygiene and restorative measures, remembering that it is only in a state of comparative health that there is any security for the permanence of the aseptic condition.

I care little for coagulants or non-coagulants. Coagulation is certain in albumen when exposed to external agencies, and all the non-coagulants in the world cannot prevent it, except by its practical destruction. I care not to exclude all organisms, for I know that to be impossible. There are many which are not pathogenic in their character. It is idle to talk of danger from anærobic, deep-seated organisms, for they are usually harmless, and if they were not they cannot be avoided. I care not that every instrument that enters the pulp chamber shall have been heated to a red heat, which is the only way completely to secure sterilization. We know that the material with which the root is filled is never made sterile, and if the manipulation is what it should be there is no necessity for it. We know that utter asepticism of the mouth is a hopeless dream, even if it were desirable. But we also know that cleanliness and dryness are essential, and we know that these are within the reach of every careful operator. We know, too, that comparative freedom from pathogenic microbes is readily attainable, and the dentist who does not secure this is unworthy the name. We know that a state of asepticism is not the only one upon which health of the tissues depends, and it is quite as excusable to neglect one necessary precaution as another. A tooth may be as radically lost through imperfect manipulation or the use of bad material as by a lack of proper medication. So I again protest against the tone of some of our modern teaching, that seems to ignore all that is not new and unheard or by the average dentist, and that affects the obscure and the occult. I am willing to sacrifice my reputation for scholarship to that of good sense. I would rather have the approval of those who are really intelligent, than the applause of any number of groundlings. If I can be known as a safe, reliable, conservative, level headed practitioner, I will resign all claims to daring, experimental, brilliant empiricism. I recognize the fact that it is necessary that there should be innovations and innovators, but I do not desire to be the patient of one. I honor those, who by patient, laborious investigation elucidate the incontestable truths of science, but I cannot give my approbation to him who catches up the half of an idea, and by an absurd extravagance manages to taint even the best cause. Therefore, while I eagerly seize upon new truths, I try to prove all things, and especially to hold fast to that which is good.—*Dental Advertiser*.

---

ARTICLE II.

### PHYSIOLOGICAL ACTION OF OBTUNDENTS.

